# Long Non-Coding RNAs Target Pathogenetically Relevant Genes and Pathways in Rheumatoid Arthritis

**DOI:** 10.3390/cells8080816

**Published:** 2019-08-02

**Authors:** Marzia Dolcino, Elisa Tinazzi, Antonio Puccetti, Claudio Lunardi

**Affiliations:** 1 Department of Medicine, University of Verona, 37134 Verona, Italy; 2 Department of Experimental Medicine—Section of Histology, University of Genova, 16132 Genova, Italy

**Keywords:** long non-coding RNA, microRNA, protein‒protein interaction network, gene module

## Abstract

Rheumatoid arthritis (RA) is a chronic inflammatory autoimmune disease driven by genetic, environmental and epigenetic factors. Long non-coding RNAs (LncRNAs) are a key component of the epigenetic mechanisms and are known to be involved in the development of autoimmune diseases. In this work we aimed to identify significantly differentially expressed LncRNAs (DE-LncRNAs) that are functionally connected to modulated genes strictly associated with RA. In total, 542,500 transcripts have been profiled in peripheral blood mononuclear cells (PBMCs) from four patients with early onset RA prior any treatment and four healthy donors using Clariom D arrays. Results were confirmed by real-time PCR in 20 patients and 20 controls. Six DE-LncRNAs target experimentally validated miRNAs able to regulate differentially expressed genes (DEGs) in RA; among them, only FTX, HNRNPU-AS1 and RP11-498C9.15 targeted a large number of DEGs. Most importantly, RP11-498C9.15 targeted the largest number of signalling pathways that were found to be enriched by the global amount of RA-DEGs and that have already been associated with RA and RA–synoviocytes. Moreover, RP11-498C9.15 targeted the most highly connected genes in the RA interactome, thus suggesting its involvement in crucial gene regulation. These results indicate that, by modulating both microRNAs and gene expression, RP11-498C9.15 may play a pivotal role in RA pathogenesis.

## 1. Introduction

Rheumatoid arthritis (RA) is an autoimmune disease characterized by chronic inflammation of the joints with severe pain and swelling, joint damage and disability, which ultimately leads to joint destruction and loss of function [1]. Several genome-wide association studies have identified genetic variants that confer RA risk. However, these variants can explain less than 20% of susceptibility in RA. Several factors have been shown to contribute to the onset of the disease, such as genetic susceptibility, environmental factors including smoking, and epigenetic mechanisms [2].

LncRNAs are epigenetic regulators of gene expression and are involved in immune and inflammatory molecular networks. Moreover, it has been demonstrated that they also play a role in several autoimmune diseases [3].

The modulation of some lncRNAs has been described in RA [4], but the interplay between lncRNAs and gene modulation in RA has only been partially explored. Indeed, only a few microarray studies described the gene expression profiling analysis of a congruous number of lncRNAs and mRNA transcripts in RA PBMCs [5,6], but in these works, only target prediction methods were used to inspect all the possible interactions among lncRNAs and coding genes. Moreover, only lncRNA‒mRNA pairs that were simply co-expressed or that are simply transcribed from nearby loci have been considered. Thus, despite these notable reports, the role of lncRNA in modulating RA pathogenesis remains unclear.

Notably, it has been demonstrated that lncRNAs can also modulate gene expression by sponging microRNAs, thus limiting the amount of miRNAs available to target mRNAs. The framework of regulatory networks must be taken into account in evaluating the potential of lncRNAs for tuning gene expression profiles.

In the present work we aimed to identify lncRNAs modulated in RA patients in order to evaluate whether they are involved in disease-associated gene modulation, only taking into account their experimentally validated gene targets. Moreover, we have analysed the complex network of molecular interactions among coding and non-coding transcripts (i.e., lncRNAs and miRNAs, that may be involved in RA pathogenesis.

## 2. Materials and Methods

### 2.1. Patients

To perform the gene array analysis we enrolled four patients, two females and two males, mean age 54 ± 15 affected by early rheumatoid arthritis, defined by the presence of positive rheumatoid factor and/or anti-citrullinated protein antibodies, of increased ESR and/or CRP, of synovitis (detected by physical examination and musculoskeletal ultrasound), lasting more than six weeks and less than six months, and by the number of small tender or swollen joints. Patients fulfilled the 2010 American College of Rheumatology/European League against Rheumatism classification criteria for RA [7]. Twelve additional patients and healthy subjects were enrolled for the RT-PCR validation test. The clinical characteristics of these patients were similar to those of the patients used for the gene array analysis. Patients were not treated with conventional disease-modifying antirheumatic drugs, biologicals, or prednisolone. Nonsteroidal anti-inflammatory drugs were allowed. Enrolled patients did not suffer from extra-articular manifestations.

Both patients and controls were subjects of Caucasian origin from Northern Italy. 

Written informed consent was obtained from all the participants to the study and the study protocol was approved by the Ethical Committee of the Azienda Ospedaliera Universitaria Integrata di Verona (identification code 1538, date of approval 23 April 2008). All the investigations have been performed according to the principles contained in the Helsinki declaration.

### 2.2. Microarray Analysis

Blood sample collection was carried out using BD Vacutainer K2EDTA tubes (Becton Dickinson, Franklin Lakes, NJ, USA) and 21-gauge needles. 

PBMCs isolation was performed by Ficoll-HyPaque (Pharmacia Biotech, Quebec, Canada) gradient centrifugation. Patients and controls had a similar PBMCs distribution. Total RNA was extracted from PBMCs (10^7^ cells) using an miRNeasy mini kit (Qiagen GmbH, Hilden, Germany). cRNA preparation, sample hybridization and scanning were performed following the protocols provided by Affymetrix (Santa Clara, CA, USA), using a Cogentech Affymetrix microarray unit (Campus IFOM IEO, Milan, Italy). All samples were hybridized on a Human Clariom D (Thermo Fisher Scientific, Waltham, MA, USA) gene chip. Signal intensities were analysed with Transcriptome Analysis Console (TAC) 4.0 software (Applied Biosystems, Foster City, CA, USA). 

Using the Human Clariom D arrays, more than 540,000 human transcripts can be interrogated, starting from as little as 100 pg of total RNA. The signal intensity was background-adjusted, normalized and log-transformed using the signal space transformation (SST)‒robust multi-array average algorithm (RMA). 

Differentially expressed genes that showed an expression level at least 1.5-fold different in the test sample versus a control sample at a significant level (FDR corrected *p*-value ≤ 0.05) were chosen for final consideration. Target annotations of long non-coding RNAs were retrieved using starBase v2.0 (http://starbase.sysu.edu.cn/starbase2/index.php), where lncRNAs interactions, experimentally validated by high-throughput experimental technologies, are registered [8].

The list of gene targets of microRNAs (miRNAs) that are targeted by lncRNAs was gathered from the FunRich database (http://www.funrich.org/) [9]. 

### 2.3. Protein‒Protein Interaction (PPI) Network Construction and Network Clustering

The PPI network was constructed upon the experimentally validated protein‒protein interactions using STRING (Search Tool for the Retrieval of Interacting Genes) version 10.5 (http://string-db.org/) [10]. Network topological analysis was performed using Cytoscape software (http://www.cytoscape.org/) [11]. High-flow areas (highly connected regions) of the network (modules) were detected using the MCODE plugin of Cytoscape (k-core = 4 and node score cutoff = 0.2).

### 2.4. Gene Functional Classification and Enrichment Analysis

Genes were functionally classified into biological processes (BPs) according to the Gene Ontology (GO) annotations (http://www.geneontology.org/) [12] by the Panther expression analysis tools (http://pantherdb.org/) [13].

Pathway classification and enrichment (Bonferroni corrected *p*-value ≤ 0.05) analysis were achieved with FunRich. 

### 2.5. Real-Time PCR of LncRNA

First, 500 ng of total RNA were treated with one unit of DNase I Amplification Grade (Invitrogen, Carlsbad, CA, USA). First-strand cDNA was generated using the SuperScript IV First-Strand Synthesis System (Invitrogen) with random hexamers, according to the manufacturer’s protocol. Real-time PCR was performed in triplicate with a PowerUp™ Sybr^®^ Green reagent (Applied Biosystems) in a QuantStudio 6 Flex system (Applied Biosystems). Transcripts’ relative expression levels were obtained after normalization against the geometric mean of the housekeeping genes GAPDH and beta-actin (ACTB) expression. The ΔΔCt method was used for comparing relative fold expression differences. Results are expressed as fold changes with respect to healthy patients.

### 2.6. Real-Time PCR of Genes Modulated in RA Patients

First-strand cDNA was obtained using the SuperScript III First-Strand Synthesis System for RT-PCR Kit (Invitrogen), with random hexamers, following the manufacturer’s protocol. PCR was performed in a total volume of 25 μL containing 1× Taqman Universal PCR Master mix, no AmpErase UNG and 2.5 μL of cDNA; pre-designed, Gene-specific primers and probe sets for each gene were obtained from Assay-on-Demand Gene Expression Products service (Applied Biosystems).

Real-time PCR reactions were carried out in a two-tube system and in singleplex. The real-time amplifications encompassed 10 min at 95 °C (AmpliTaq Gold activation), followed by 40 cycles at 95 °C for 15 s and at 60 °C for 1 min. Thermocycling and signal detection were performed with a 7500 Sequence Detector (Applied Biosystems). Signals were detected by following the manufacturer’s instructions. This methodology allows for the identification of the cycling point where the PCR product is detectable by means of fluorescence emission (threshold cycle or Ct value). The Ct value correlates to the quantity of target mRNA. Relative expression levels were calculated for each sample after normalization against the housekeeping genes GAPDH, beta-actin and 18s ribosomal RNA (rRNA), using the ΔΔCt method for comparing relative fold expression differences. Ct values for each reaction were determined using TaqMan SDS analysis software (Applied Biosystems). For each amount of RNA tested, triplicate Ct values were averaged. Since Ct values vary linearly with the logarithm of the amount of RNA, this average represents a geometric mean.

### 2.7. Real-Time PCR of MicroRNA 

miRNA expression was evaluated by TaqMan^®^ Advanced miRNA assays chemistry (Applied Biosystems). Briefly, 10 ng of total RNA was reverse transcribed and pre-amplified with TaqMan^®^ Advanced miRNA cDNA synthesis kit according to the manufacturer’s instructions (Applied Biosystems). Pre-amplified cDNA was diluted 1/10 in nuclease-free water and 5 µL of diluted cDNA for each replicate were loaded in PCR. 20 µL PCR reactions were composed by 2× Fast Advanced Master Mix and TaqMan^®^ Advanced miRNA assay for miR-520e. The mean of Ct for hsa-miR-16-5p and hsa-miR-26a-5p expression was used to normalize miRNA expression. Real-time PCR was carried out in triplicate on a QuantStudio 6 Flex instrument (Applied Biosystems). Expression values were reported as fold change with respect to healthy controls by the ΔΔCt method, employing QuantStudio Real-Time PCR system software v. 1.3.

### 2.8. Statistical Analysis

Statistical testing was performed using SPSS Statistics 2 software (IBM, Armonk, NY, USA). Data obtained from RT-PCR analysis of RA samples and healthy controls were analysed using the Mann‒Whitney Test.

## 3. Results

### 3.1. High-Throughput Gene and Long Non-Coding RNA Expression Profiling in Peripheral Blood Mononuclear Cells of RA Patients

We simultaneously profiled the expression of more than 540,000 human transcripts, including those ascribed to more than 50,000 long non-coding RNAs (lncRNAs), in four PBMC samples from patients with clinically diagnosed RA symptoms, with the purpose of identifying lncRNAs potentially involved in RA pathogenesis. RA-associated transcriptional profiles were compared to those obtained from four age- and sex-matched healthy subjects and, 97 lncRNAs and 942 coding genes were selected applying a robust filtering approach (FDR-corrected *p*-value ≤ 0.05 and fold change ≥ |1.5|) (Tables S1 and S2).

The functional classification by Gene Ontology (http://www.geneontology.org/) of the 942 differentially expressed genes (DEGs) highlighted the modulation of transcripts that play a role in biological processes (BPs) strictly associated with RA, including apoptosis, cell proliferation, cell migration, inflammatory response, immune response, angiogenesis, extracellular matrix degradation and bone resorption. In particular, we observed that the bone resorption BP included upregulated genes that negatively regulate osteoblast functions, such as HES1, and genes that are involved in osteoclast development, like ATP6AP1, TCTA and SBNO2 [14,15,16]. In this regard, we also have to mention the upregulation of CSF1/MG-CSF that is one of the most important soluble factors responsible for osteoclast maturation and survival [17]. Interestingly, PLCB1, a positive regulator of osteoblast differentiation [18], was downregulated in RA samples.

Several DEGs were involved in well-known pathways including Wnt, TNF, type I interferon, p38 MAP kinase, NF-kB, Toll-like receptors, Jak-Stat, PI3K and mTOR signalling that have already been associated with RA pathogenesis. A selection of genes involved in the abovementioned functional classes is given in Table 1 and Table 2.

All the differentially expressed transcripts were submitted to a pathway enrichment analysis that highlighted other meaningful signalling networks in which modulated genes were involved. These pathways included, for example, signalling that operates in vascular biology (i.e., PAR, uPA/uPAR, PDGFR, endothelins and VEGF signalling), interferon-gamma, EGF-receptor, Arf6, IL-5, IL-3 and S1P1 signalling pathways. All the enriched pathways (Bonferroni corrected *p*-value ≤ 0.05) are listed in Table 3.

### 3.2. Selected Long Non-Coding RNAs Modulated in RA Patients Have the Potential to Regulate Genes Differentially Expressed in the Disease

To strengthen the significance of our analysis, we interrogated the StarBase database to select only modulated lncRNAs for which experimentally validated microRNA (miRNA) targets had already been annotated and, by this criterion, six out of 97 lncRNAs were filtered: FTX, HNRNPU-AS1, MIATNB, RP11-498C9.15, RP4-714D9.5 and RP11-73E17.2. All these lncRNAs were downregulated except RP11-498C9.15 and RP11-73E17.2, which were overexpressed in RA samples (Table 4). 

To find all the possible interactions among modulated genes and the selected lncRNAs, we verified if they could target miRNAs able to regulate RA-DEGs. We therefore analysed the complete list of genes regulated by the miRNA targets of the six lncRNA that were validated by high-throughput technologies, and selected only those microRNAs that could modulate differentially expressed genes in RA patients. We thus observed that all the selected lncRNAs, via their miRNA targets, could control genes differentially expressed in RA patients, but only FTX, HNRNPU-AS1 and RP11-498C9.15 targeted quite a large number of DEGs (Table 4 and Table S3).

To gain insight on the potential role played by the selected lncRNAs in regulating gene clusters that were most probably associated with the disease pathogenesis, we performed a pathways enrichment analysis of all their targeted DEGs. This approach led us to observe that genes targeted by RP11-498C9.15 significantly enriched (Bonferroni *p*-value < 0.05) the largest number of signalling pathways and, interestingly, these pathways were almost the same as those that were globally enriched by the 942 modulated genes (Table 5). On the contrary, FTX and HNRNPU-AS1 targeted a small number of enriched pathways that were also targeted by RP11-498C9.15, whereas MIATNB RP4-714D9.5 and RP11-73E17.2 did not target any enriched pathways (Table S4). These results led us to suppose that RP11-498C9.15 may exert a major impact on gene modulation associated with RA so, to examine its possible involvement in the disease pathogenesis, we focused our analysis on genes targeted by this lncRNA.

**Figure 1:**
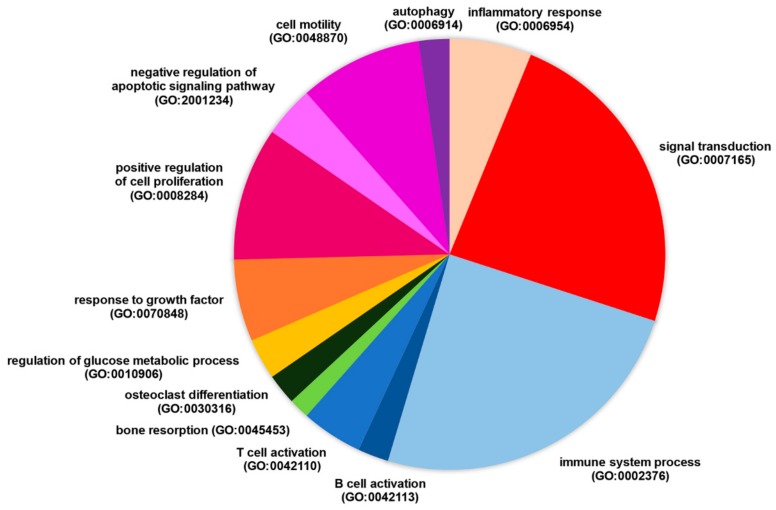
Biological processes involving modulated genes targeted by RP11-498C9.15 in patients with RA.

We observed that several DEGs that were targeted by RP11-498C9.15 were involved in meaningful biological settings, such as the immune and inflammatory response, bone metabolism, apoptosis regulation, etc. (Figure 1). Moreover, the modulation of several of them has already been associated with RA. Upregulated targeted genes were, for example, implicated in T cell survival (DUSP5) [19] or activation (i.e., MAL, LAT and CD81) [20,21,22], whereas others were involved in B cell development (i.e., PRDM1/BLIMP-1, MEF2D, LRRC8A and ZBTB7A) [23,24,25]. Notably, CD81 has been found to be upregulated in RA synoviocytes [26], while single-nucleotide polymorphisms of the gene BLIMP-1 have already been described in RA [23].

A fair number of targeted genes played a role in the inflammatory response, like DDIT4/REDD-1, COTL1/CLP, SPATA2, PTGER4, PTGES2, NR4A2, LTB and KLF2. Among these, the pro-inflammatory gene DDIT4 has a role in NF-kB activation [27], which has been shown to modulate the production of inflammatory cytokines implicated in RA joint pathology [28]. SPATA2 is a component of the TNF-alpha signalling [29], while COTL1 is able to regulate the production of leukotriene A4 [30] and NR4A2 and LTB are highly expressed in inflamed RA synovial tissues [31,32]. The gene product of PTGES2 converts prostaglandin H2 to prostaglandin E2, whereas PTGER4 encode for the prostaglandin E2 receptor and, interestingly, this molecule is involved in the differentiation and expansion of T helper lymphocytes, a process that is involved in RA onset [33]. In addition, this molecule exerts an inhibitory action on human bone marrow stromal cells-mediated bone matrix mineralization [34].

Notably, other upregulated targeted genes included transcripts that are involved in bone erosion, like CRTC2, a negative regulator of BMP2-induced osteogenic cell differentiation [35], and JUNB, involved in osteoclast development [36] and strongly expressed in RA fibroblast-like cells [37]. In addition, it has been demonstrated that JUNB promotes Th17 cells’ development, but inhibits T regulatory cells’ fate during chronic autoimmunity [38].

We also observed that RP11-498C9.15 targeted upregulated genes involved in the negative control of the apoptotic process (i.e., SH3BGRL3 and WEE1) and in autophagy (i.e., ATG4D), a mechanism that seems to be implicated in apoptosis resistance in RA as well as in the development of several autoimmune diseases including RA [39]. Interestingly, RP11-498C9.15 targeted FOXJ3 and gene polymorphism of this transcript have been associated with RA [40].

### 3.3. LncRNA RP11-498C9.15 Targets RA-Associated Meaningful Signalling Pathways

Since, as mentioned above, DEGs targeted by RP11-498C9.15 enriched signalling pathways that were also over-represented in the whole RA dataset, we analysed the entire list of these molecular networks (Table 5) and found that almost all are involved in pathogenetic mechanisms that may play a role in the development of RA and, interestingly, that the activation of several of them has already been associated with RA pathogenesis. Indeed, the involvement of phosphatidylinositide-3-kinase (PI-3K), AKT, mTOR and sphingosine-1 phosphate (S1P1) signalling in RA pathogenesis has been extensively documented [41,42]. 

The most enriched pathway was the beta-1 integrin signalling, a cluster of molecules that are well represented at the synovial lining layer, where synovial cells adhere to cartilage. It has been observed that the RA-associated pro-inflammatory milieu promotes the synthesis of beta-1 integrins [43] and indeed, fibroblast, macrophages and endothelial cells of RA synovial tissue express high levels of these molecules. Notably, the beta-1 integrin binding to laminin at the synovial lining, strongly increases the expression of metalloproteases [43] and signalling by beta-1 integrins leads to immune cell activation, stimulates cell migration, cytokine production and new vessels formation. Besides the integrins pathway, interferon-gamma was the second most enriched signalling—not surprising since this cytokine is rapidly accumulated in RA synovial tissue by activated T cells and its level of expression correlates with the RA radiographic severity [44].

Pathways involved in vascular biology were also significantly enriched in DEGs modulated by RP11-498C9.15, including “PAR1-mediated thrombin signalling events,” “urokinase-type plasminogen activator (uPA)/uPAR-mediated signalling,” and “endothelins” signalling; indeed, it is well known that RA patients have generalized vasculopathy and finger blood flow abnormalities.

A high level of thrombin activity has been found in RA patients, and it has been suggested that this molecule may have a strong mitogenic effect on synovial fibroblast-like cells, thus possibly playing a significant role in RA pathogenesis. Moreover, it has been observed that the high levels of thrombin that have been detected in RA synovium are associated with increased expression of platelet derived growth factor beta (PDGF-b) [45] and, interestingly, its pathway was also enriched. Remarkably, PDGF receptor activation promotes the RA synoviocytes’ pro-destructive behaviour [46].

RA synovial fibroblasts were reported to express high levels of plasminogen activator and urokinase (uPA), and a pro-inflammatory role has been hypothesized for this molecule, since anti-inflammatory glucocorticoids strongly suppress uPA gene expression [47]. Finally, endogenous endothelins are involved in articular inflammation by modulating inflammatory pain, oedema formation and leukocyte migration. In addition, an elevated plasma level of endothelin-1 has been observed in RA, which may be associated with the symptoms of vascular dysregulation frequently observed in RA patients [48].

RP11-498C9.15 also targeted members of the proteoglycan glypican and syndecan pathways, which are structural molecules thought to govern cell migration, tissue invasion and angiogenesis [49], thus possibly playing a crucial role in fibroblast-like synoviocytes’ behaviour [50]. Other enriched pathways involved in angiogenesis were the signalling of focal adhesion kinases (FAKs), epidermal growth factor (EGF) receptor and VEGF/VEGF-receptor. The former has been implicated in both RA inflammatory angiogenesis and in RA synovial fibroblasts’ pro-invasive activity [51,52], whereas the second is particularly involved in synovial fibroblast proliferation. Indeed, EGF and EGFR serum concentrations are increased in RA patients compared to healthy subjects [53]. VEGF also has pro-inflammatory and bone-destructive skills and its level correlates with the RA disease activity [54].

It has been described that RA osteoclasts exhibit increased activity and, interestingly, pathways involved in bone erosion were also significantly enriched, including Arf6, as well as hepatocyte growth factor (HGF) signalling. The Arf6 pathway plays an important role in osteoclast maturation [55], while HGF promotes osteoclast activation and inhibits osteoblast differentiation, thus favouring bone-erosive processes. Interestingly, plasma levels of this cytokine can predict joint damage in RA [56].

RP11-498C9.15 also targeted enriched pathways involved in glucose metabolism like “insulin” and “insulin growth factor (IGF1)” signalling. The activation of these pathways may reflect the abnormal glucose metabolism that characterizes a good percentage of RA patients [57] and has been correlated with the degree of systemic inflammation [58]. In addition, it has been observed that the pro-inflammatory cytokine TNF may promote insulin resistance by phosphorylation of the insulin receptor [59].

Enriched targeted pathways also included the granulocyte-macrophage colony-stimulating factor (GM-CSF) signalling, a cytokine highly expressed in both RA synovial fluid and tissue and on circulating mononuclear cells from RA patients. Given its prominent role in macrophage differentiation and activation, it has been suggested that GM-CSF inhibition may represent a favourable therapeutic approach to treat RA. Indeed, early phases of clinical trials evaluating anti-GM-CSF therapy demonstrated its potential clinical benefit in RA patients [60].

Finally, other enriched targeted pathways included interleukin-5 (IL-5) and interleukin-3 (IL-3) signalling. IL-5 is known to stimulate B cells’ growth, increasing immunoglobulin secretion, whereas IL-3 is a potent inducer of RANKL expression in human basophils and an important role for this molecule in the early phase of collagen-induced arthritis has been demonstrated [61].

### 3.4. LncRNA RP11-498C9.15 Targets Highly Connected Genes in the RA Transcriptome

Since it is well known that the targeting of highly connected genes can have a more pronounced impact on the development of a disease than the modulation of transcripts that show no functional interactions, we wanted to verify that RP11-498C9.15 could control highly interacting genes in the RA transcriptome.

**Figure 2:**
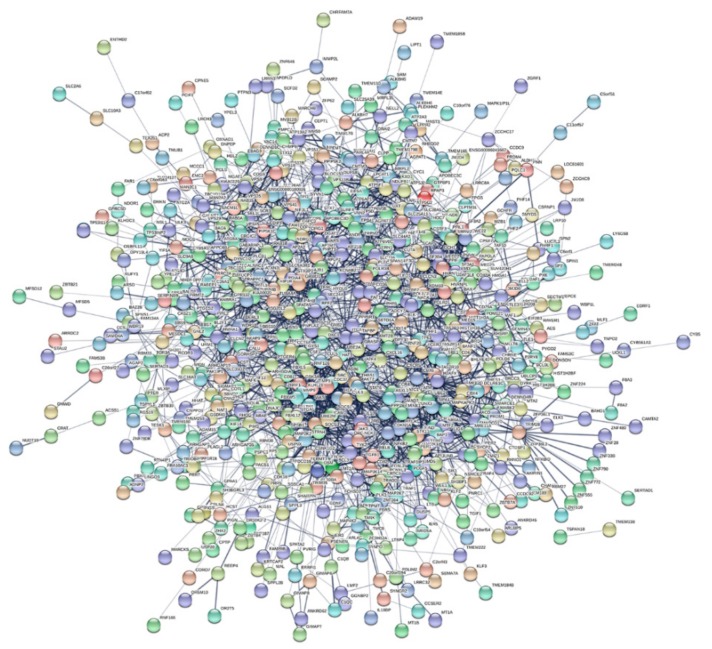
Protein‒protein interaction (PPI) network of genes modulated in RA patients.

**Figure 3:**
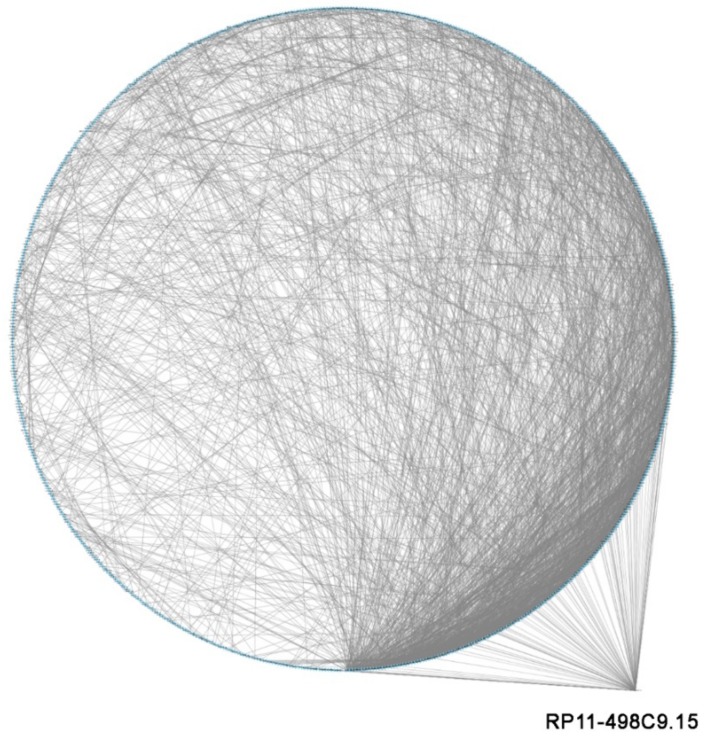
Graphical representation of connections among RP11-498C9.15 and modulated genes in the PPI network. Differentially expressed genes in RA patients are ordered around a circle based on their degree of connectivity (number of edges).

With this purpose in mind, we first built a protein‒protein interaction (PPI) network that included all the experimentally validated functional interactions among the protein products of the 942 modulated genes in RA; thereafter, we performed a modular analysis to find the areas of the network in which the most highly connected genes were clustered. The obtained network included 755 nodes (genes) and 2496 edges (pairs of interactions) and exhibited a good enrichment *p*-value (*p* < 1.0e^−16^) (Figure 2). Interestingly, the topological analysis of the PPI network revealed that, via their miRNAs targets, RP11-498C9.15 was connected to genes with a high degree of connectivity (Figure 3), whereas the modular analysis highlighted the presence of six modules (Table S5) that included genes regulated by RP11-498C9.15 (Table 6).

In module M1, CUL3, HNRNPA0 and SOCS3 were targeted and, notably, the latter has been found to be upregulated in RA fibroblast-like synoviocytes and RA PBMCs [62]. In module M2, RP11-498C9.15 targeted ANO6, which is involved in bone mineralization, whereas in module M3, the lncRNA targeted ATP5G2, TERF2 and the abovementioned PTGES2, which, as previously mentioned, operates the conversion of prostaglandin H2 to prostaglandin E2.

Module M4 included seven targeted genes, ATM, NACA, PCGF5, SEL1L, SURF4, THBS1 and VPS45, while in module M5, AKT2, JUND, RELA and SRC were targeted. In this regard, we have to mention that AKT2 induces the proliferation and migration of RA fibroblast-like synoviocytes [63], JUND can contribute to bone erosion [64], RELA is a key component of the NF-kB transcription factor crucially involved in RA [65], and SRC has bone resorptive properties.

In module M6, STX12, which is involved in autophagy, was targeted.

The level of expression of RP11-498C9.15, selected miRNA and gene targets were validated by RT-PCR (Figure S1). Statistically significant differences between patients and healthy subjects were found in the expression levels of all the tested transcripts.

## 4. Discussion

RA is an inflammatory chronic autoimmune disease and its pathogenesis is influenced by genetic, environmental and epigenetic factors [1]. LncRNAs are key components of the epigenetic machinery that can regulate chromatin remodelling and gene expression by interacting with other epigenetic factors and with genes. Interestingly, lncRNAs seem to be involved in the development of several autoimmune diseases [3].

In the present work, we simultaneously profiled a large number of coding and non-coding transcripts in the same cohort of early-phase-RA patients in order to identify the lncRNAs that most probably shape the outcomes of crucial biological processes strictly associated with RA pathogenesis.

The criteria adopted to select deregulated lncRNAs in RA rely on the analysis of experimentally validated functional interaction among coding and non-coding partners of the RA transcriptome. Using this approach, six lncRNAs were filtered; such lncRNAs, via their miRNA targets, can modulate differentially expressed genes in RA patients.

Since it is now a common notion that disease can be explained in terms of molecular pathways’ perturbation, we performed a pathway enrichment analysis of all modulated genes that were targeted by the six selected lncRNAs. Through this analysis we observed that transcripts targeted by RP11-498C9.15, unlike transcripts targeted by the other five lncRNAs, showed a good coverage of pathways significantly modulated in the disease. Indeed, RP11-498C9.15 was able to target almost all the signalling pathways in which genes modulated in RA samples are involved, thus showing the best correlation to the RA transcriptome. 

Notably, RP11-498C9.15 targeted meaningful signalling pathways that have been already associated with RA and RA-fibroblast-like synoviocytes’ behaviours, while the other selected lncRNAs targeted only a few pathways that were also modulated by RP11-498C9.15.

By analysing the whole pattern of functional interactions among the modulated genes, we could observe that RP11-498C9.15 targeted modules of the most highly connected genes in the RA interactome, which are believed to be principally involved in the disease onset.

Also, for this reason, although we cannot exclude the involvement of the other selected lncRNAs, we believe that RP11-498C9.15 may play a crucial role in the pathogenesis of RA.

We are aware that a limitation of this work is the small number of samples analysed, but this is mainly due to the difficulty of recruiting RA patients in the early phase of the disease and in the absence of any treatment.

We believe that, especially if our results are confirmed on a larger cohort of patients, the lncRNA RP11-498C9.15 deserves to be identified as a candidate in the design of novel therapeutic strategies in RA.

## Figures and Tables

**Table 1:** Selected biological processes in which genes modulated in RA are involved.

ID	Fold Change	FDR *p*-Value	Gene Symbol	Description	mRNA Accession
**Apoptosis**					
TC0300009843.hg.1	2.12	0.050	TP63	tumour protein p63	NM_001114978
TC2200007783.hg.1	5.34	0.008	PIM3	Pim-3 proto-oncogene, serine/threonine kinase	NM_001001852
TC0300010770.hg.1	12.5	0.008	CSRNP1	cysteine-serine-rich nuclear protein 1	NM_033027
TC1900009429.hg.1	2.3	0.018	KHSRP	KH-type splicing regulatory protein	NM_003685
TC0600011441.hg.1	3.47	0.008	BAG6	BCL2-associated athanogene 6	NM_001199697
TC1100013230.hg.1	2.08	0.023	BCL9L	B-cell CLL/lymphoma 9-like	NM_182557
TC0100007449.hg.1	2.2	0.049	SH3BGRL3	SH3 domain binding glutamate-rich protein like 3	NM_031286
TC1100006815.hg.1	3	0.037	WEE1	WEE1 G2 checkpoint kinase	NM_001143976
**Cell proliferation**					
TC0300009702.hg.1	2.49	0.044	EIF4G1	eukaryotic translation initiation factor 4 gamma, 1	NM_001194946
TC0X00007132.hg.1	3.18	0.008	CDK16	cyclin-dependent kinase 16	NM_001170460
TC0200015764.hg.1	3.79	0.004	CNPPD1	cyclin Pas1/PHO80 domain containing 1	NM_015680
TC0200016494.hg.1	2.04	0.046	CNNM4	cyclin and CBS domain divalent metal cation transport mediator 4	NM_020184
TC1900010696.hg.1	5.13	0.007	AKT2	v-akt murine thymoma viral oncogene homolog 2	NM_001243027
**Cell migration**					
TC1500010018.hg.1	2.3	0.018	SEMA7A	semaphorin 7A, GPI membrane anchor	NM_001146029
TC1200012859.hg.1	3.65	0.006	RHOF	ras homolog family member F (in filopodia)	NM_019034
TC1100009864.hg.1	3.29	0.004	RHOG	ras homolog family member G	NM_001665
TC1700009528.hg.1	2.46	0.028	CXCL16	chemokine (C-X-C motif) ligand 16	NM_001100812
**Inflammatory response**					
TC1500006925.hg.1	24.36	0.018	THBS1	thrombospondin 1	NM_003246
TC1900006977.hg.1	11.9	0.050	ICAM1	intercellular adhesion molecule 1	NM_000201
TC0500007231.hg.1	3.28	0.022	PTGER4	prostaglandin E receptor 4 (subtype EP4)	NM_000958
TC0100011384.hg.1	2.52	0.025	MAPKAPK2	mitogen-activated protein kinase-activated protein kinase 2	NM_004759
TC0100009364.hg.1	3.82	0.008	CSF1	colony stimulating factor 1 (macrophage)	NM_000757
TC0300006985.hg.1	6.3	0.034	CCR4	chemokine (C-C motif) receptor 4	NM_005508
TC1900010743.hg.1	2.7	0.019	TGFB1	transforming growth factor beta 1	NM_000660
TC1000007990.hg.1	4.92	0.020	DDIT4	DNA damage inducible transcript 4	NM_019058
TC1600011060.hg.1	2.28	0.017	COTL1	coactosin-like F-actin binding protein 1	NM_021149
TC2000009401.hg.1	3.54	0.011	SPATA2	Spermatogenesis-associated 2	NM_00113577
TC1100011243.hg.1	4.25	0.007	RELA	v-rel avian reticuloendotheliosis viral oncogene homolog A	NM_001145138
TC0900011623.hg.1	2.16	0.030	PTGES2	prostaglandin E synthase 2	NM_001256335
TC0200014672.hg.1	7.65	0.042	NR4A2	nuclear receptor subfamily 4, group A, member 2	NM_006186
TC1900007270.hg.1	6.2	0.007	KLF2	Kruppel-like factor 2	NM_016270
TC1700011903.hg.1	4.41	0.046	SOCS3	suppressor of cytokine signalling 3	NM_003955
TC1600009395.hg.1	3.76	0.010	SOCS1	suppressor of cytokine signalling 1	NM_003745
TC0100017107.hg.1	2.71	0.041	IL10	interleukin 10	NM_000572
TC1100009225.hg.1	7.04	0.006	CXCR5	chemokine (C-X-C motif) receptor 5	NM_001716
TC1100013178.hg.1	2.22	0.023	MAP4K2	mitogen-activated protein kinase kinase kinase kinase 2	NM_001307990
TC1700007262.hg.1	4.43	0.023	MAP2K3	mitogen-activated protein kinase kinase 3	NM_002756
TC1900009325.hg.1	3.56	0.038	MAP2K2	mitogen-activated protein kinase kinase 2	NM_030662
TC0600011438.hg.1	2.07	0.045	LTB	lymphotoxin beta (TNF superfamily, member 3)	NM_002341
TC1700011903.hg.1	4.41	0.046	SOCS3	suppressor of cytokine signalling 3	NM_003955
**Immune response**					
TC0200008452.hg.1	2.85	0.024	MAL	mal, T-cell differentiation protein	NM_002371
TC1100006576.hg.1	3.74	0.015	CD81	CD81 molecule	NM_001297649
TC0600007495.hg.1	2.08	0.037	HLA-A	major histocompatibility complex, class I, A	NM_001242758
TC0100007291.hg.1	2.08	0.028	C1QC	complement component 1, q subcomponent, C chain	NM_001114101
TC1100007787.hg.1	3.29	0.035	CD6	CD6 molecule	NM_001254750
TC1600011368.hg.1	3.14	0.008	LAT	linker for activation of T-cells	NM_001014987
TC1900008279.hg.1	5.58	0.006	BCL3	B-cell CLL/lymphoma 3	NM_005178
TC1900008166.hg.1	3.13	0.004	CD79A	CD79a molecule, immunoglobulin-associated alpha	NM_001783
TC1200010950.hg.1	2.47	0.034	STAT6	signal transducer and activator of transcription 6, interleukin-4 induced	NM_001178078
TC1000008891.hg.1	10.97	0.008	DUSP5	dual specificity phosphatase 5	NM_004419
TC0600008972.hg.1	5.51	0.018	PRDM1	PR domain containing 1, with ZNF domain	NM_001198
TC0100016000.hg.1	4.15	0.004	MEF2D	myocyte enhancer factor 2D	NM_001271629
TC0900008891.hg.1	2.21	0.023	LRRC8A	leucine rich repeat containing 8 family, member A	NM_001127244
TC1900009320.hg.1	2.71	0.045	ZBTB7A	zinc finger and BTB domain containing 7A	NM_015898
TC1900008505.hg.1	2.93	0.018	BAX	BCL2-associated X protein	NM_001291428
TC1800007805.hg.1	2.22	0.027	NFATC1	nuclear factor of activated T-cells, cytoplasmic, calcineurin-dependent 1	NM_001278669
TC2200008637.hg.1	3.64	0.015	IL2RB	interleukin 2 receptor, beta	NM_000878
**Angiogenesis**					
TC1200010839.hg.1	2	0.029	ITGA5	integrin alpha 5	NM_002205
TC2000007336.hg.1	2.83	0.015	PPP1R16B	protein phosphatase 1, regulatory subunit 16B	NM_001172735
TC1600008971.hg.1	2.83	0.018	JMJD8	jumonji domain containing 8	NM_001005920
TC1700011818.hg.1	2.6	0.050	JMJD6	jumonji domain containing 6	NM_001081461
TC0100007832.hg.1	17.12	0.008	ZC3H12A	zinc finger CCCH-type containing 12A	NM_025079
TC0100018300.hg.1	2.44	0.032	ADAM15	ADAM metallopeptidase domain 15	NM_001261464
**Bone resorption**					
Positive regulation of bone resorption					
TC0X00008831.hg.1	2.62	0.021	ATP6AP1	ATPase, H+ transporting, lysosomal accessory protein 1	NM_001183
TC2000007283.hg.1	2.11	0.026	SRC	SRC proto-oncogene, non-receptor tyrosine kinase	NM_005417
TC0300009916.hg.1	4.17	0.030	HES1	hes family bHLH transcription factor 1	NM_005524
TC0100015891.hg.1	2.76	0.008	CRTC2	CREB regulated transcription coactivator 2	NM_181715
TC1900010009.hg.1	3.41	0.023	JUND	jun D proto-oncogene	NM_001286968
**Positive regulation of osteoclast proliferation/differentiation**					
TC0300007380.hg.1	2.4	0.027	TCTA	T-cell leukaemia translocation altered	NM_022171
TC1900009134.hg.1	9.56	0.002	SBNO2	strawberry notch homolog 2	NM_014963
TC0100009364.hg.1	3.82	0.008	CSF1	colony stimulating factor 1 (macrophage)	NM_000757
TC0X00008831.hg.1	2.62	0.021	ATP6AP1	ATPase, H+ transporting, lysosomal accessory protein 1	NM_001183
TC0300009916.hg.1	4.17	0.030	HES1	hes family bHLH transcription factor 1	NM_005524
TC0500007231.hg.1	3.28	0.022	PTGER4	prostaglandin E receptor 4 (subtype EP4)	NM_000958
TC1900007096.hg.1	5.16	0.032	JUNB	jun B proto-oncogene	NM_002229
**Osteoblast differentiation**					
TC2000009887.hg.1	−2.8	0.0172	PLCB1	phospholipase C, beta 1 (phosphoinositide-specific)	NM_015192
**Extracellular matrix degradation**					
TC2000007514.hg.1	3.14	0.041	MMP9	matrix metallopeptidase 9	NM_004994
TC0500012599.hg.1	1.9	0.040	ADAM19	ADAM metallopeptidase domain 19	NM_033274
TC0100018300.hg.1	2.44	0.032	ADAM15	ADAM metallopeptidase domain 15	NM_001261464
TC1900006470.hg.1	3.01	0.011	BSG	basigin	NM_001728

**Table 2:** Selected signalling pathways involving genes modulated in RA.

ID	Fold Change	FDR *p*-Value	Gene Symbol	Description	mRNA Accession
**Wnt signalling pathway**					
TC1900009272.hg.1	2.4	0.018	AES	amino-terminal enhancer of split	NM_001130
TC1100008181.hg.1	2.03	0.029	LRP5	LDL-receptor-related protein 5	NM_001291902
TC0200014672.hg.1	7.65	0.042	NR4A2	nuclear receptor subfamily 4, group A, member 2	NM_006186
TC1100007913.hg.1	2.69	0.048	MARK2	MAP/microtubule affinity-regulating kinase 2	NM_001039469
TC1200007595.hg.1	2.88	0.023	SMARCD1	SWI/SNF-related, matrix-associated, actin-dependent regulator of chromatin, subfamily d, member 1	NM_003076
TC1900011639.hg.1	2.1	0.032	STK11	serine/threonine kinase 11	NM_000455
TC1100008181.hg.1	2.03	0.029	LRP5	LDL-receptor-related protein 5	NM_001291902
**TNF signalling pathway**					
TC0900011385.hg.1	−1.99	0.040	PSMD5	proteasome 26S subunit, non-ATPase 5	NM_001270427
TC0600011438.hg.1	2.07	0.045	LTB	lymphotoxin beta (TNF superfamily, member 3)	NM_002341
TC1700010879.hg.1	2.36	0.026	MAP3K14	mitogen-activated protein kinase kinase kinase 14	NM_003954
TC1600010616.hg.1	2.14	0.035	TRADD	TNFRSF1A-associated via death domain	NM_003789
TC1100011243.hg.1	4.25	0.007	RELA	v-rel avian reticuloendotheliosis viral oncogene homolog A	NM_001145138
**Type I interferon signalling**					
TC0600007495.hg.1	2.08	0.037	HLA-A	major histocompatibility complex, class I, A	NM_001242758
TC0200008452.hg.1	2.85	0.024	MAL	mal, T-cell differentiation protein	NM_002371
TC0100017107.hg.1	2.71	0.041	IL10	interleukin 10	NM_000572
TC1600009395.hg.1	3.76	0.010	SOCS1	suppressor of cytokine signalling 1	NM_003745
TC1100011243.hg.1	4.25	0.007	RELA	v-rel avian reticuloendotheliosis viral oncogene homolog A	NM_001145138
TC1900009627.hg.1	2.63	0.040	TYK2	tyrosine kinase 2	NM_003331
TC1000008727.hg.1	2.64	0.024	NFKB2	nuclear factor of kappa light polypeptide gene enhancer in B-cells 2 (p49/p100)	NM_001077494
**p38 MAP kinase signalling**					
TC1400009524.hg.1	2.91	0.029	ZFP36L1	ZFP36 ring finger protein-like 1	NM_001244698
TC1700007262.hg.1	4.43	0.023	MAP2K3	mitogen-activated protein kinase kinase 3	NM_002756
TC0100011384.hg.1	2.52	0.025	MAPKAPK2	mitogen-activated protein kinase-activated protein kinase 2	NM_004759
TC0X00009581.hg.1	2.99	0.013	ELK1	ELK1, member of ETS oncogene family	NM_001114123
TC0100016000.hg.1	4.15	0.004	MEF2D	myocyte enhancer factor 2D	NM_001271629
**NF-kB signalling pathway**					
TC1900008300.hg.1	2.78	0.034	RELB	v-rel avian reticuloendotheliosis viral oncogene homolog B	NM_006509
TC1900008279.hg.1	5.58	0.006	BCL3	B-cell CLL/lymphoma 3	NM_005178
TC1600010616.hg.1	2.14	0.035	TRADD	TNFRSF1A-associated via death domain	NM_003789
TC1100011243.hg.1	4.25	0.007	RELA	v-rel avian reticuloendotheliosis viral oncogene homolog A	NM_001145138
TC1700010879.hg.1	2.36	0.026	MAP3K14	mitogen-activated protein kinase kinase kinase 14	NM_003954
TC0800012190.hg.1	3.65	0.018	SHARPIN	SHANK-associated RH domain interactor	NM_030974
**TOLL-like receptors signalling pathways**					
TC0200008452.hg.1	2.85	0.024	MAL	mal, T-cell differentiation protein	NM_002371
TC0X00009581.hg.1	2.99	0.013	ELK1	ELK1, member of ETS oncogene family	NM_001114123
TC0100011384.hg.1	2.52	0.025	MAPKAPK2	mitogen-activated protein kinase-activated protein kinase 2	NM_004759
TC1000008727.hg.1	2.64	0.024	NFKB2	nuclear factor of kappa light polypeptide gene enhancer in B-cells 2	NM_001077494
TC1700007262.hg.1	4.43	0.023	MAP2K3	mitogen-activated protein kinase kinase 3	NM_002756
TC1100011243.hg.1	4.25	0.007	RELA	v-rel avian reticuloendotheliosis viral oncogene homolog A	NM_001145138
**Jak-Stat signalling pathway**					
TC1600009395.hg.1	3.76	0.010	SOCS1	suppressor of cytokine signalling 1	NM_003745
TC1700011903.hg.1	4.41	0.046	SOCS3	suppressor of cytokine signalling 3	NM_003955
TC1900009991.hg.1	2.63	0.042	JAK3	Janus kinase 3	NM_000215
TC1200010950.hg.1	2.47	0.034	STAT6	signal transducer and activator of transcription 6, interleukin-4 induced	NM_001178078
**PI3K signalling pathway**					
TC1900010696.hg.1	5.13	0.007	AKT2	v-akt murine thymoma viral oncogene homolog 2	NM_001243027
TC1100008136.hg.1	2.31	0.023	RPS6KB2	ribosomal protein S6 kinase, 70kDa, polypeptide 2	NM_003952
TC1000007990.hg.1	4.92	0.020	DDIT4	DNA damage inducible transcript 4	NM_019058
TC1000008891.hg.1	10.97	0.008	DUSP5	dual specificity phosphatase 5	NM_004419
TC0700008560.hg.1	3.76	0.026	GNB2	guanine nucleotide binding protein (G protein), beta polypeptide 2	NM_005273
TC1900010009.hg.1	3.41	0.023	JUND	jun D proto-oncogene	NM_001286968
TC1900007096.hg.1	5.16	0.032	JUNB	jun B proto-oncogene	NM_002229
TC0600008972.hg.1	5.51	0.018	PRDM1	PR domain containing 1, with ZNF domain	NM_001198
**mTOR signalling pathway**					
TC0300013684.hg.1	2.21	0.043	TFRC	transferrin receptor	NM_001128148
TC1200007595.hg.1	2.88	0.023	SMARCD1	SWI/SNF-related, matrix-associated, actin-dependent regulator of chromatin, subfamily d, member 1	NM_003076
TC1700011903.hg.1	4.41	0.046	SOCS3	suppressor of cytokine signalling 3	NM_003955
TC2000007283.hg.1	2.11	0.026	SRC	SRC proto-oncogene, non-receptor tyrosine kinase	NM_005417
TC0400009765.hg.1	3.06	0.018	MXD4	MAX dimerization protein 4	NM_006454
TC0100016000.hg.1	4.15	0.004	MEF2D	myocyte enhancer factor 2D	NM_001271629

**Table 3:** Signalling pathways significantly enriched in genes modulated in RA patients.

Biological Pathway	Bonferroni Corrected *p-*Value
Proteoglycan syndecan-mediated signalling events	0.006
Alpha9 beta1 integrin signalling events	0.020
GMCSF-mediated signalling events	0.025
Beta1 integrin cell surface interactions	0.026
IL3-mediated signalling events	0.027
IFN-gamma pathway	0.028
PAR1-mediated thrombin signalling events	0.031
Thrombin/protease-activated receptor (PAR) pathway	0.032
Syndecan-1-mediated signalling events	0.032
Integrin family cell surface interactions	0.032
Plasma membrane oestrogen receptor signalling	0.033
Endothelins	0.040
Signalling events mediated by focal adhesion kinase	0.040
PDGFR-beta signalling pathway	0.040
Arf6 trafficking events	0.040
Class I PI3K signalling events mediated by Akt	0.040
mTOR signalling pathway	0.040
Internalization of ErbB1	0.040
EGF receptor (ErbB1) signalling pathway	0.040
Class I PI3K signalling events	0.040
Arf6 signalling events	0.040
ErbB1 downstream signalling	0.040
Arf6 downstream pathway	0.040
Insulin Pathway	0.040
Urokinase-type plasminogen activator (uPA) and uPAR-mediated signalling	0.040
S1P1 pathway	0.040
EGFR-dependent Endothelin signalling events	0.041
IGF1 pathway	0.044
ErbB receptor signalling network	0.045
Sphingosine 1-phosphate (S1P) pathway	0.045
IL5-mediated signalling events	0.045
Signalling events mediated by hepatocyte growth factor receptor (c-Met)	0.047
PDGF receptor signalling network	0.047
Nectin adhesion pathway	0.049
Signalling events mediated by VEGFR1 and VEGFR2	0.051
Glypican 1 network	0.056
Glypican pathway	0.055
VEGF and VEGFR signalling network	0.055
Integrin-linked kinase signalling	0.053

**Table 4:** Selected lncRNAs modulated in RA patients.

ID	Fold Change	FDR *p*-Value	Gene Symbol	mRNA Accession	miRNA Targets	Targeted Modulated Genes
TC0X00010064.hg.1	−2.03	0.049	FTX	NR_028379	64	96
TC0100018570.hg.1	−2.64	0.034	HNRNPU-AS1	NR_026778	55	161
TC2200009240.hg.1	−2	0.042	MIATNB	NR_110543	16	11
TC1700012077.hg.1	2.5	0.039	RP11-498C9.15	ENST00000582866.1	27	106
TC0100009198.hg.1	−3.23	0.008	RP4-714D9.5	ENST00000564623.1	6	27
TC1400006883.hg.1	2.74	0.018	RP11-73E17.2	ENST00000557373.1	1	4

**Table 5:** Signalling pathways enriched in RA-DEGs that are targeted by RP11-498C9.15.

Biological Pathway	Bonferroni Corrected *p-*Value
Beta1 integrin cell surface interactions	0.004
Integrin family cell surface interactions	0.006
IFN-gamma pathway	0.008
PAR1-mediated thrombin signalling events	0.008
Thrombin/protease-activated receptor (PAR) pathway	0.009
Plasma membrane oestrogen receptor signalling	0.009
Endothelins	0.009
Glypican pathway	0.014
Proteoglycan syndecan-mediated signalling events	0.016
Signalling events mediated by focal adhesion kinase	0.030
Class I PI3K signalling events mediated by Akt	0.030
Internalization of ErbB1	0.030
Arf6 downstream pathway	0.030
Arf6 trafficking events	0.030
EGF receptor (ErbB1) signalling pathway	0.030
Urokinase-type plasminogen activator (uPA) and uPAR-mediated signalling	0.030
ErbB1 downstream signalling	0.030
S1P1 pathway	0.030
Arf6 signalling events	0.030
mTOR signalling pathway	0.030
Insulin pathway	0.030
PDGFR-beta signalling pathway	0.030
Class I PI3K signalling events	0.030
EGFR-dependent Endothelin signalling events	0.030
IGF1 pathway	0.031
GMCSF-mediated signalling events	0.031
IL5-mediated signalling events	0.031
Signalling events mediated by hepatocyte growth factor receptor (c-Met)	0.032
PDGF receptor signalling network	0.032
IL3-mediated signalling events	0.032
Nectin adhesion pathway	0.032
Signalling events mediated by VEGFR1 and VEGFR2	0.033
Glypican 1 network	0.034
Syndecan-1-mediated signalling events	0.035
VEGF and VEGFR signalling network	0.036
Alpha9 beta1 integrin signalling events	0.037
Sphingosine 1-phosphate (S1P) pathway	0.040
ErbB receptor signalling network	0.040
Integrin-linked kinase signalling	0.045

**Table 6:** Modulated genes included in the six modules that are targeted by RP11-498C9.15.

Module	miRNAs	Gene
M1	hsa-miR-23c (1.56 up)	CUL3
	hsa-miR-23b-3p (2.06 up)	CUL3
	hsa-miR-23a-3p (1.74 down)	CUL3
	hsa-miR-221-3p (2.34 up)	HNRNPA0
	hsa-miR-302c-3p (2.48 down)	SOCS3
	hsa-miR-221-3p (2.34 up)	SOCS3
M2	hsa-miR-372-3p (1.63 up)	ANO6
M3	hsa-miR-101-3p (1.80 up)	ATP5G2
	hsa-miR-137 (2.54 up)	PTGES2
	hsa-miR-101-3p (1.80 up)	TERF2
	hsa-miR-613 (1.55 down)	TERF2
	hsa-miR-221-3p (2.34 up)	TERF2
	hsa-miR-206 (2.04 up)	TERF2
M4	hsa-miR-4735-3p (2.53 up)	ATM
	hsa-miR-101-3p (1.80 up)	NACA
	hsa-miR-137 (2.54 up)	PCGF5
	hsa-miR-101-3p (1.80 up)	SEL1L
	hsa-miR-101-3p (1.80 up)	SURF4
	hsa-miR-613 (1.55 down)	THBS1
	hsa-miR-4735-3p (2.53 up)	THBS1
	hsa-miR-221-3p (2.34 up)	THBS1
	hsa-miR-206 (2.04 up)	THBS1
	hsa-miR-18b-5p (1.67 down)	THBS1
	hsa-miR-18a-5p (2.32 up)	THBS1
	hsa-miR-613 (1.55 down)	VPS45
	hsa-miR-206 (2.04 up)	VPS45
M5	hsa-miR-137 (2.54 up)	AKT2
	hsa-miR-613 (1.55 down)	JUND
	hsa-miR-206 (2.04 up)	JUND
	hsa-miR-520e (1.83 down)	RELA
	hsa-miR-520d-3p (1.92 down)	RELA
	hsa-miR-520c-3p (1.52 down)	RELA
	hsa-miR-520b (1.70 down)	RELA
	hsa-miR-520a-3p (1.50 down)	RELA
	hsa-miR-372-3p (1.63 up)	RELA
	hsa-miR-302e (2.18 down)	RELA
	hsa-miR-302d-3p (1.84 down)	RELA
	hsa-miR-302c-3p (2.48 down)	RELA
	hsa-miR-302b-3p (1.66 down)	RELA
	hsa-miR-302a-3p (1.58 down)	RELA
	hsa-miR-137 (2.54 up)	SRC
M6	hsa-miR-23c (1.56 up)	STX12
	hsa-miR-23b-3p (2.06 up)	STX12
	hsa-miR-23a-3p (1.74 down)	STX12
	hsa-miR-206 (2.04 up)	STX12

## Supplementary Materials

The following are available online at https://www.mdpi.com/2073-4409/8/8/816/s1, Table S1: Genes modulated in RA patients versus healthy subjects, Table S2: LncRNAs modulated in RA patients versus healthy subjects, Table S3: Modulated genes in RA patients that are targeted by miRNA targets of the selected lncRNAs, Table S4: Enriched signalling pathways involving modulated genes that are targeted by the selected lncRNAs, Table S5: Modulated genes in RA patients that are included in the six modules, Figure S1: Expression of selected genes, RP11-498C9.15 and miR-520e in RA patients versus healthy subjects. Bars indicate SD. The histograms represent the mean of the results obtained in 20 healthy donors and in 20 RA patients. The Mann‒Whitney test was used to compare the two groups’ means.
