# Supplementary figures and images for: Long Non-Coding RNAs Target Pathogenetically Relevant Genes and Pathways in Rheumatoid Arthritis
Source: Cells. 2019 Aug 2;8(8):816. doi: 10.3390/cells8080816 (PMC6721587; doi:10.3390/cells8080816)

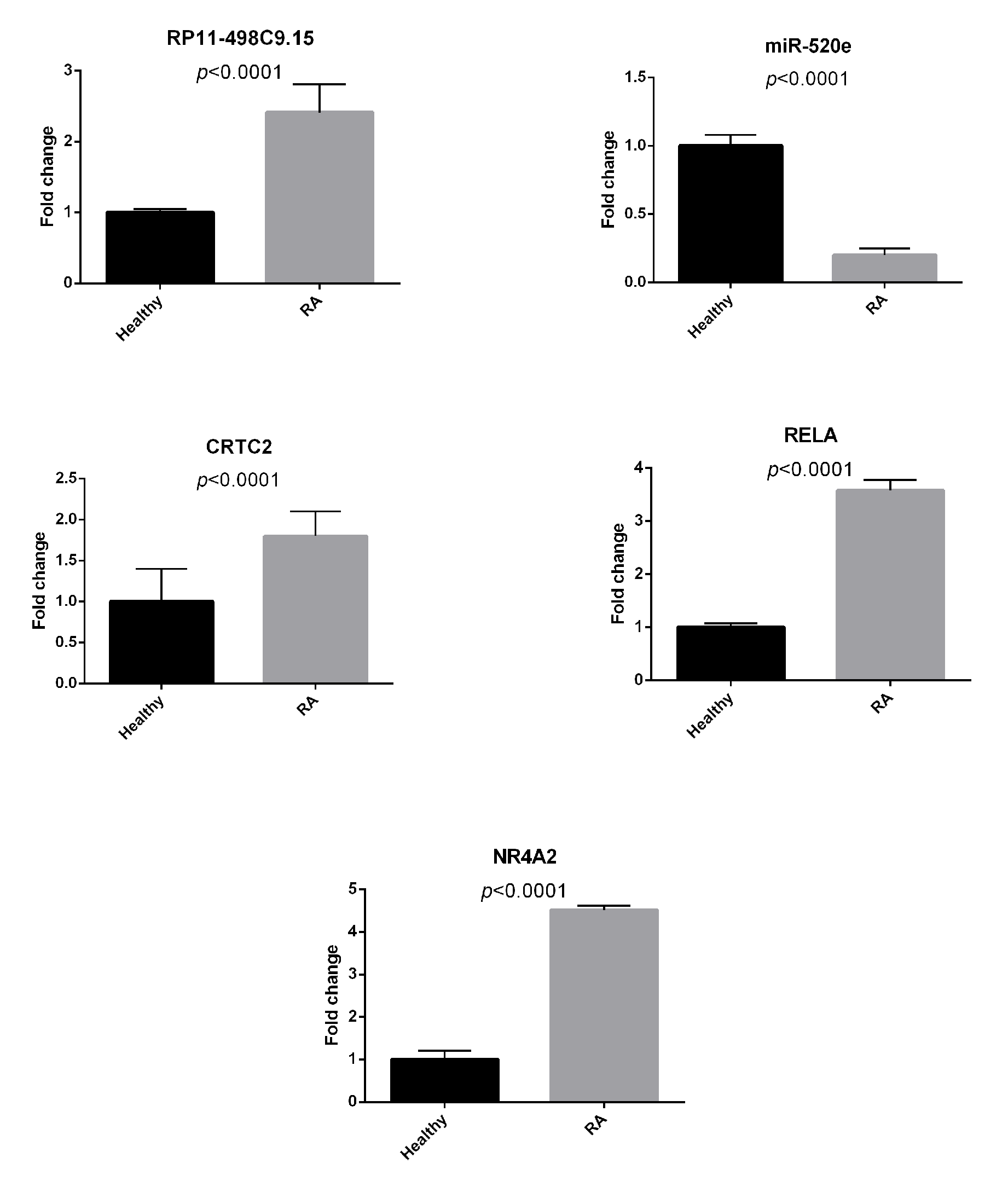

Supplement: Supplementary file 1 [file cells-08-00816-s001.zip › Figure S1_RA.tif]
